# Case of Acute Atrophy of the Liver

**Published:** 1853-03

**Authors:** Chas. A. Lee

**Affiliations:** Buffalo


### BUFFALO MEDICAL JOURNAL

AND

### MONTHLY REVIEW.

VOL. 8. MARCH, 1853. NO. 10.

### ORIGINAL COMMUNICATIONS.

ART. I.—*Case of Acute Atrophy of the Liver.* By CHAS. A. LEE, M. D.

Mary Berry, Irish, aged 22, servant, generally robust and healthy, entered the Hospital of the Sisters of Charity, of Buffalo, Jan. 21st, 1853. Has been somewhat unwell for several weeks; troubled with nausea and vomiting, and occasionally diarrhœa; seems inclined to coma; but little power over her limbs; skin slightly icterode, and temperature of skin about natural; face considerably bloated; limbs somewhat œdematous; pulse slow and feeble; tongue furred; disposed to lie on her back. A mustard plaster was prescribed, to be applied to epigastrium, and a teaspoonful of a mixture composed of three drops of creasote and ℥ij water, every hour, to allay the sickness and vomiting. In addition, a pill of ½ gr. opium, and ij grs. blue mass, was added every six hours.

*Jan. 24th.* Pulse 76. Tongue thickly furred. The skin yellow, bordering, in some places, on a dusky-brown; heat of skin about natural. The sickness of stomach had been entirely relieved by the creasote and mustard; bowels had not been moved since her entrance. Inclined to sleep constantly, but says she feels better.

*Jan. 25th.* Pulse 68. Tongue continues furred; countenance sullen; very stupid. Temperature of skin natural. No pain. No movement of bowels. Increased yellowness of skin. Prescribed *hyd. sub. mur.* gr. x, *rhei*, gr. x, *ol. menth.* gtt. iij, followed by *Rochelle salts*, *senna* and *anise seed*, a wine glass of a strong solution, every hour till it should operate.

*Jan.* 26*th.* The stomach had rejected most of the medicine. No operation. Pulse 88. Heat of skin natural. Tongue furred and dry. Much thirst; inclines to coma, but can be roused. No urine secreted, nor has she passed any since her admittance, as can be ascertained. Skin deep-yellow, or bronze color, over whole body. The right hypochondriac region was directed to be rubbed with an ointment, consisting of equal parts of *compound iodine* and *mercurial ointment*, and a stimulating enema administered.

*Jan.* 27*th.* About the same. Still comatose. Directed warm *brandy toddy*, with grs. v, *carb. ammonia*, every two hours, and a drop of *Croton oil* every two hours, between same, till it should operate.

*Jan.* 28*th.* The coma increased; one or two involuntary evacuations had taken place from the bowels; the pulse became feeble and more frequent; and she expired toward morning.

*Autopsy ten hours after death.*

All the tissues were deeply tinged of a bright yellow hue. Nothing abnormal was noticed in any of the organs, except the liver, which was placed in the hands of Prof. John C. Dalton, who has kindly favored me with the following account of its appearance:

JANUARY 28, 1853.

This liver was reduced to one half its natural size, and very flabby and loose in consistency. The exterior was in parts pale, but mostly of a dusky hue, considerably darker than the normal color of the organ. There was no opacity or other inflammatory alteration of its peritoneal covering. The organ presented the appearances designated by Rokitansky, under the name of "Acute Atrophy of the Liver." The cut surface showed a considerable portion in a state of partial hyperæmia, the capillaries in the centre of each acinus being gorged with blood, while the intervening spaces remained of a yellowish color; giving the marbled appearance to the cut surface, known as "nutmeg liver." There were, also, large portions in which the whole tissue was so infiltrated with bright yellow biliary matter, that though the form of the acini was preserved, all distinction between these component parts was lost; and the hepatic tissue presented the appearance of a congeries of vessels injected with yellow paint. The portions in which this alteration had taken place were mostly globular in shape, and varied from the size of a hazel-nut to that of half an egg. They were considerably more friable than the rest of the hepatic tissue. When situated near the exterior, these portions projected somewhat above the surface of the organ, presenting an appearance very similar to that of a mass of varicose veins, except for the strong yellow color, which showed distinctly through the peritoneal coat.

The bile contained in the gall bladder, was very black and tarry in consistency. J. C. D.

*Remarks.* — This affection appears to have been first described by Rokitansky, and is still unnoticed in our standard works on practical medicine. He described it as "Yellow Atrophy of the Liver," an acute affection, "the expression of a constitutional malady," and having its immediate origin in anomalies of the blood. It is characterized by the saturated yellow color, owing to a diffusion of bile throughout the tissue, by extreme flabbiness and pulpiness, loss of the granular texture, extreme rapidity in the reduction of size, which chiefly affects the vertical diameter and consequently induces a flattening of the liver. He remarks that it occurs chiefly in the early years of life, during puberty and middle age, and is remarkable for the rapid course it runs, for extreme tenderness of the liver, nervous attacks and jaundice, and that it terminates fatally with febrile symptoms of a disorganized state of the blood, irritation of the brain and its membranes, hydrocephalic softening of the former, and with symptoms of exudation and suppuration generally, and especially of the mucous membrane, pneumonia, &c.

He also calls attention to the fact that the blood contained in the large vessels of the liver and in the trunk of the vena porta, is reduced in consistence, and of a dirty reddish-brown color, and that the coats of the latter vessel are tinged with bile; and this, he remarks, points to the fact that the portal blood itself contains such an excess of biliary constituents, that they are separated here, and still more in the capillaries, and thus fill the entire vascular and biliary system; the coats of the vessels and the cellular strata thus absorb bile by exosmosis, the true glandular tissue fuses, is lost in the biliary colliquation and disappears. The immediate consequences of this condition are, that the blood in the vena cava is infected and overcharged with bile, causing intense jaundice, and when this has reached a certain point the above symptoms terminate in a rapid consumption of the blood and in exhaustion, and it is not unusual to find in these cases, biliary matter of a deep-yellow color, or a black tarry substance in the intestines, owing to an exudation of the disorganized blood through the mucous membrane. This affection is readily distinguished from Lænnec's *Cirrhosis*, which is a chronic affection, though the color of the liver resembles that of acute yellow atrophy, but instead of being soft and flabby, the liver is abnormally firm and tough.

There is a similar affection described by Rokitansky, called "*Red Atrophy of the Liver*," which is distinguished from the yellow by its dark-brown, or bluish-red color; the liver is gorged with blood, and presents a spongy, elastic consistency; there is an absence of granulation, and the texture is perfectly homogeneous when cut into; the organ is reduced in size, though its thickness preponderates over the other dimensions.

It is, moreover, a chronic affection, and accompanied by torpor of the abdominal ganglia, venous plethora of the abdominal viscera, and by the formation of brownish-black, or greenish-black, tarry bile, an fæces of a similar constitution. Rokitansky thinks that death generally results in this latter disease, from the congestion of the portal system.

We have only met with one well-marked case of this disease before, and that was many years since in dispensary practice, in New York. The symptoms were very similar to those above described in Mrs. Berry. That it is not very uncommon is very probable, as Prof. Dalton informs me that he saw two or three cases of it the last summer, while attending the autopsies of Rokitansky, in the General Hospital, at Vienna. The symptoms, as it will have been noticed, were, with the exception of jaundice, very similar to those produced by the retention of urea in the blood; and as I could not ascertain the state of the urinary secretion, it is very possible that the comatose symptoms may have been partly due to that cause. The coma and jaundice, however, increased pari-passu, until death took place. Rokitansky, it seems, attributes death in this disease to a "rapid consumption of the blood, and exhaustion, consequent on overcharging the blood with bile."

We submit this case to the profession, with the hope that the attention of others may be directed to this disease, who have better opportunities of observation. There is no class of diseases involved in more obscurity, at this present time, than those of the liver; and none need more the patient investigation of practiced and enlightened pathologists. Our country opens a wide scope for such study; but to be successful, the investigation is not to be limited to the liver only, but extended to the constitution of the blood, and the various modifications, consequent on the different kinds of hepatic derangement.

BUFFALO, February 10, 1853.

